# Dynamic Information-Hiding Method with High Capacity Based on Image Interpolating and Bit Flipping

**DOI:** 10.3390/e25050744

**Published:** 2023-05-01

**Authors:** Cheng-Ta Huang, Cheng-Yi Lin, Chi-Yao Weng

**Affiliations:** 1 Department of Information Management, Yuan Ze University, Taoyuan 32003, Taiwan; cthuang2020@saturn.yzu.edu.tw; 2 International Bachelor Program in Informatics, Yuan Ze University, Taoyuan 32003, Taiwan; s1083518@mail.yzu.edu.tw; 3 Department of Computer Sciences and Artificial Intelligence, National Pingtung University, Pingtung 90003, Taiwan

**Keywords:** neighbor mean interpolation (NMI), ones’ complement, flipped data, high capacity

## Abstract

In this era of rapid information exchange in public networks, there is a risk to information security. Data hiding is an important technique for privacy protection. Image interpolation is an important data-hiding technique in image processing. This study proposed a method called neighbor mean interpolation by neighboring pixels (NMINP) that calculates a cover image pixel by neighbor mean interpolation and neighboring pixels. To reduce image distortion, NMINP limits the number of bits when embedding secret data, making NMINP have a higher hiding capacity and peak signal-to-noise ratio (PSNR) than other methods. Furthermore, in some cases, the secret data are flipped, and the flipped data are treated in ones’ complement format. A location map is not needed in the proposed method. Experimental results comparing NMINP with other state-of-the-art methods show that NMINP improves the hiding capacity by more than 20% and PSNR by 8%.

## 1. Introduction

With advanced communication networks, information will be continuously transported through private or public networks. This makes it convenient for people to store or search for data. Behind this convenience, there are many risks. The advancement of communication networks also comes at a time when privacy concerns are on the rise. Information security is, therefore, a critical issue. No matter how well you protect data through a secure transmission, they may be hacked by others. Nevertheless, one possible solution is to improve data transmission security, and data hiding is a technique for effectively addressing this issue. Data hiding is the process of embedding data into a piece of an image or video to achieve the function of not being tampered with arbitrarily. The goal of data hiding is to obtain a high hiding capacity (HC) and peak signal-to-noise ratio (PSNR).

There are many existing data-hiding methods, which can be categorized into transform [1,2], encryption [3], spatial [4,5,6,7], and compressed domains [8,9,10]. In the transform domain, a high frequency for data hiding is used because high frequencies are less sensitive to human eyes. Examples of state-of-the-art methods under this category include discrete cosine transform [1] and discrete wavelet transform [2]. In the encryption domain [3], the cover image is encrypted, and secret data can be embedded in the encrypted image pixels. An example of the spatial domain is the least significant bit (LSB) replacement [4,5]; however, LSB replacement and shifting is not safe (does not pass RS analysis). Under this domain, two other techniques have been proposed: histogram shifting (HS) [6] and difference expansion [7]. In HS, the point between the zero point and the peak of histograms is shifted to embed data. Difference expansion embeds data in the expanded difference of neighboring pixels. In the compressed domain, compressed image codes serve as a carrier for the secret data, which can be embedded in them at a lower compression ratio. AMBTC [8], JPEG [9], and VQ [10] are examples of compressed image formats.

Data-hiding technology can also be divided into two parts depending on whether it can restore the original image or not: reversible data hiding (RDH) and non-reversible data hiding (NRDH). RDH can be divided into five categories in the spatial domain: HS [6], pixel value ordering [11,12], interpolation technology [13,14,15,16], difference expansion [7], and prediction error expansion [17]. In 2006, histogram shifting was proposed, which can achieve the highest lower bound of PSNR with a higher hiding capacity. In 2013, pixel value ordering was proposed. It is based on prediction error expansion, so it can reduce the number of shifting pixels to maintain a better image quality. Choosing the flat block to embed data can improve the performance of embedding and embedding the same number of data can obtain a higher PSNR than other methods. In 2003, difference expansion was proposed, using the difference between the pixels of an image to embed the secret data, and the stego-image can be restored to the original image. This method can achieve a high embedding capacity and keep the distortion lower. In 2009, prediction error expansion was proposed, which is a data-hiding method based on the modification of prediction errors to solve the problem that the embedding capacity of existing histogram shifting is limited by the frequency of the most common pixel. In 2009, Jung et al. [13] proposed the first neighbor mean interpolation (NMI), with a high HC and visual quality. In 2012, Lee et al. [14] proposed the first interpolation by neighboring pixels on the maximum difference. The benefits of this method are a high embedding capacity with good image quality. In 2015, Hu et al. [15] proposed a method that increases the HC by the maximum difference between neighboring pixels. This method also has low complexity and a good image quality. In 2020, Chen et al. [16] proposed an efficient data-hiding method based on image interpolation. This method can be adopted into four different interpolation techniques: NMI, interpolation by neighboring pixels, enhanced NMI, and new interpolation expansion. When embedding the secret data, the original image pixel will be taken as a reference. However, for interpolation-based data-hiding methods, if the difference between the cover and the original image pixels is less than two, the pixel will not embed any secret data, reducing the HC. Furthermore, if the HC is increased, the quality of the stego-image decreases.

Therefore, in this paper, to solve the problem of information security, a data-hiding method, NMI by neighboring pixels (NMINP), which increases the HC and image quality after embedding the secret data based on NMI, ones’ complement, and LSB, is proposed. The proposed method is adapted from the methods previously proposed by Jung et al. [13] and Chen et al. [16]. The results provide a comparison with other methods, including those of Jung et al. [13], Chen et al. [16], and Malik et al. [18]. Different from their methods, in the proposed method, NMI generates cover image pixels without directly embedding the secret data. Taking the original image pixel as a reference, the cover image pixels are closer to the original image pixels than when the general NMI is used. Before embedding the secret data, the complexity of the image is calculated to determine the maximum number of secret data that will be embedded into each pixel. However, there is a limitation depending on the first bit of the secret data and the bits to be embedded: each pixel can embed at most the number of bits according to the complexity and at least one bit. The pixel embeds the number of bits according to the complexity, transforming the secret data into ones’ complement format before embedding. When the pixel embeds less than the number of bits calculated by the complexity, the secret data are added or subtracted from the cover image pixel. If the pixel embeds one bit, the secret data are embedded in the cover image pixel by using LSB replacement. Furthermore, the location map is not used to record other information in the proposed method, meaning the hiding capacity of our method is higher than other methods.

The rest of this paper is organized as follows. In Section 2, related interpolation-based data-hiding methods and works are introduced. Section 3 introduces the framework and algorithm of the proposed method. Furthermore, a simple example of data-embedding and extraction is illustrated in detail. Section 4 shows the experimental results, including the performance of the proposed method, comparisons of HC, and visual analyses. Finally, the conclusion is presented in Section 5.

## 2. Related Work

In this section, some related data-hiding techniques and mathematical methods, including NMI, LSB, and bit flipping, are introduced. Image interpolation was used in the proposed method.

### 2.1. Least Significant Bit (LSB)

Chan et al. [4] first proposed the technique based on LSB in 2004. It provides the simplest method of embedding secret data. This LSB-based method embeds data in the rightmost bit of the original image pixel, slightly distorting the pixel. This LSB-based data-hiding technique uses Equation (1) to obtain the stego-image pixel:

*S* = *O* − (*O* mod 2*^K^*) + *D*, (1)

where *O* and *S* represent the pixels of the original and stego-images, respectively, *D* represents the secret bit, and *K* represents the number of secret bits, *D,* that will be embedded.

Equation (2) is used to extract the secret bit:

*D* = *S* mod 2*^K^* (2)

**Figure 1:**
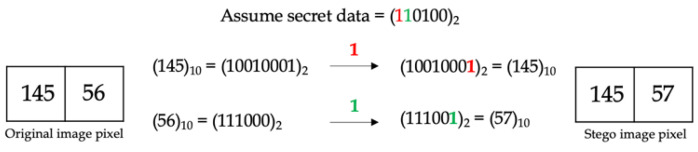
Embedding 1-bit secret data using LSB.

**Figure 2:**
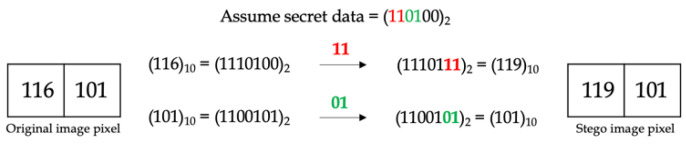
Embedding 2-bit secret data using LSB.

Figure 1 shows examples of embedding 1-bit secret data using the LSB technique, whereas Figure 2 shows the embedding of 2-bit secret data.

### 2.2. Bit Flipping

The method of bit flipping is typically used in ones’ complement operation. With the number in binary format, if the bit is 1, after applying ones’ complement, it changes to 0. If the bit is 0, it changes to 1. Bit flipping was used in the proposed method to enable it to record any other information about the secret data without using a location map. Here, the secret data are transformed into their ones’ complement when the first bit of embedding secret data is 0. For example, if the secret data embed (00101)_2_, the receiver can extract only (101)_2_. The receiver will not know whether there is 0 before (101)_2_. By using ones’ complement to generate each cover image pixel, the secret data can be embedded with 1 as the first bit, limiting the number of embedding bits. Thus, the receiver can judge whether the secret data are transformed from ones’ complement.

### 2.3. First Neighbor Mean Interpolation Method

**Figure 3:**
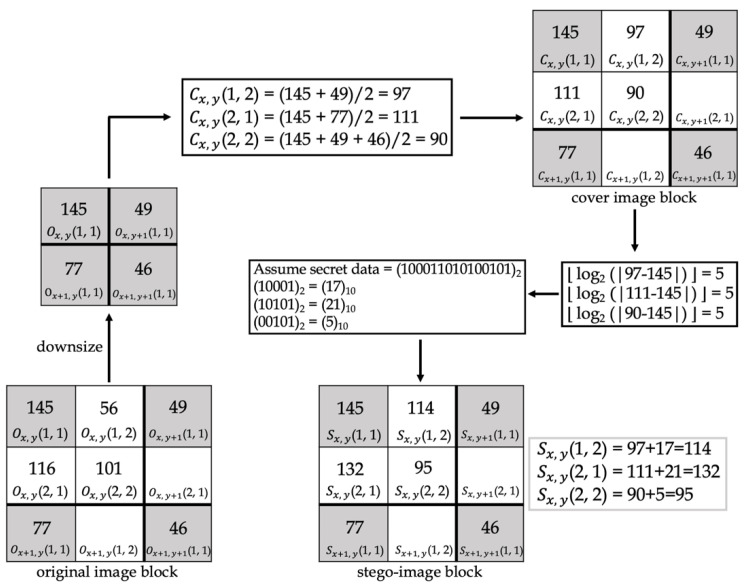
Example of neighbor mean interpolation (NMI).

Jung et al. [13] proposed the first NMI technique in 2009. Figure 3 demonstrates this method. Considering an *M × M*-sized original image, this image is downsized into an (*M*/2) × (*M*/2)-sized image, which is then divided into 2 × 2 non-overlapping blocks, with each block positioned from left to right and up to down at (1, 1), (1, 2), (2, 1), and (2, 2), respectively. Next, NMI is applied to scale-up the downsized image into an *M* × *M*-sized cover image. The cover image pixel is subtracted from the pixel in location (1, 1) in the log and floor function to calculate how many secret bits are to be embedded in the pixel. Changing the secret bits from a decimal format to a binary format, the cover image pixel is added to the secret data to obtain the stego-image pixel. The original image pixel is denoted by $O_{x,\;y}(i,\;j)$, the cover image pixel is denoted by $C_{x,\;y}(i,\;j)$, where *x* and *y* range from 1 to *M*, and (*i, j*) denotes the location of the pixel in the block. When calculating the pixels in one block (*x*, *y*), three pixels are used: $O_{x,\;y}(1,\;1),$ $O_{x,\;y+1}(1,\;1),$ and $O_{x+1,\;y}\left(1,\;1\right)$. The cover image pixel can be produced using Equation (3):

(3) $$\left\{\begin{array}{c} C_{x,\;y}(1,1)=O_{x,\;y}(1,1)\\ C_{x,\;y}(1,2)=[O_{x,\;y}(1,1)+O_{x,\;y+1}(1,1)]/2\\ C_{x,\;y}(2,1)=[O_{x,\;y}(1,1)+O_{x+1,\;y}(1,1)]/2\\ C_{x,\;y}(2,2)=[O_{x,\;y}(1,1)+O_{x,\;y+1}(1,1)+O_{x+1,\;y}(1,1)]/3 \end{array}\right.$$

### 2.4. An Efficient Data-Hiding Method Based on Image Interpolation

**Figure 4:**
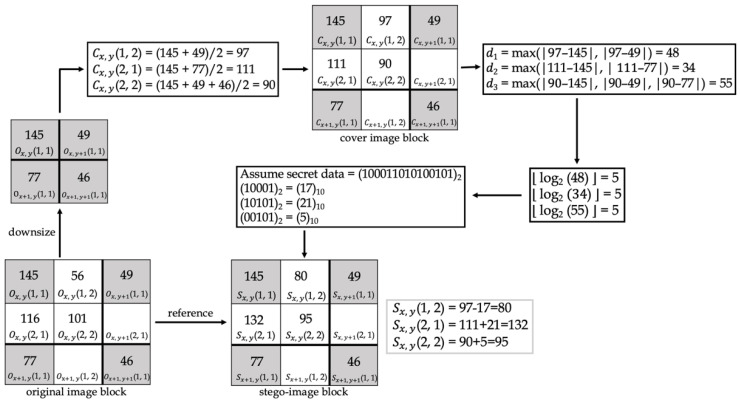
Example of Chen et al.’s [16] method.

Chen et al. [16] proposed an efficient data-hiding method based on image interpolation in 2020. Figure 4 demonstrates this method. This method differs from the method proposed by Jung et al. [13] in the subsequent operations after NMI produces the cover image. First, calculate the difference (*d*). The difference uses the log and floor function to calculate how many secret bits are to be embedded in the pixel. The secret bits are transformed from a decimal format into a binary format before embedding. When embedding the secret data, the original pixel will be taken as a reference. If the cover image pixel is smaller than or equal to the original image pixel, the cover image pixel is added to the secret data to obtain the stego-image pixel. If the cover image pixel is larger than the original image pixel, the cover image pixel is subtracted from the secret data to obtain the stego-image pixel.

### 2.5. Differences between Other Methods and the Proposed Method

The main differences between the other methods and the proposed method are shown in Table 1. In the proposed method, no additional information location map is required. The proposed method does not require additional space to record the location map. The embedding procedure of the proposed method is dynamic to achieve a high embedding capacity. We also used the original image as a reference to reduce the chance of overflow and underflow. For a 2 × 2 pixels block, 3 pixels can be used to embed secret data.

## 3. Proposed Scheme

**Figure 5:**
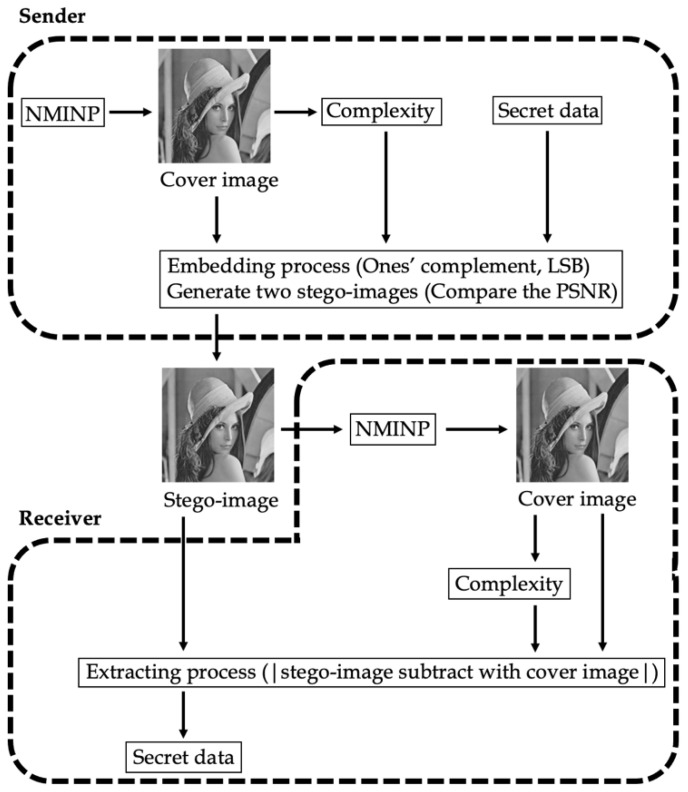
The overall flowchart of the proposed method.

In this section, the framework of the proposed method based on interpolation (NMINP) is introduced. NMINP is divided into two parts: data-embedding and extraction. Figure 5 shows the overall flowchart of the proposed method. As shown in Figure 5, the proposed method, NMINP, was used to obtain the cover image, and the complexity of the cover image was also calculated. Considering the cover image, complexity, and secret data, the proposed embedding method was used to embed the secret data into the cover image. After the embedding process, two stego-images were generated. Then, the stego-image with the best result was selected as the final stego-image. On the receiver side, the receiver can use NMINP to obtain the cover image and extract information from the stego-image using the LSB method. Then, the secret data were extracted based on the complexity of the cover image. More details of the algorithm will be explained in Section 3.1, Section 3.2, Section 3.3, Section 3.4, Section 3.5, Section 3.6, Section 3.7 and Section 3.8.

**Figure 6:**
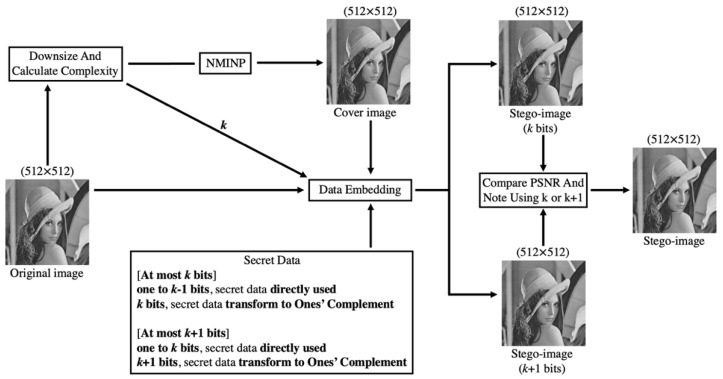
Framework of data-embedding in the proposed scheme.

Figure 6 shows the framework of data-embedding. First, the original image was downsized, and the complexity of the downsized image was calculated. The complexity calculation determines the greatest number of bits that will be embedded in each pixel, denoted by *k*, and calculated as *k* + 1. Second, the cover image was produced by NMINP. Third, to obtain two stego-images with different greatest numbers of embedded bits in each pixel, one was embedded with at most *k* bits in each pixel, while the other one was embedded with at most *k* + 1 bits in each pixel, and each pixel in the two stego-images will embed at least 1 bit. For the stego-image which embedded at most *k* bits in each pixel, if the first bit of secret data is 1, the pixel can embed 1 to *k* − 1 bits of secret data, and the secret data will be directly embedded. If the first bit of secret data is 0, the pixel can embed *k* bits of secret data and transform it to ones’ complement format before embedding. For the stego-image which embedded at most *k* + 1 bits in each pixel, if the first bit of secret data is 1, the pixel can embed 1 to *k* bits of secret data, and the secret data will be directly embedded. If the first bit of secret data is 0, the pixel can embed *k +* 1 bits of secret data and transform it to ones’ complement format before embedding. Limiting the number of bits improves the HC, and NMINP does not need to use the location map to record which secret data bits begin from 0. After calculating the secret data that will be embedded in each pixel, the original image was taken as a reference when embedding the secret data. When the original image pixel is larger than or equal to the cover image pixel, the cover image pixel is added to the secret data; otherwise, the cover image pixel is subtracted from the secret data. Then, two stego-images were produced. The last step was to compare the PSNR of these two stego-images, and if the PSNR difference is less than or equal to 1.5, the stego-image which is embedded with at most *k* + 1 bits in each pixel was chosen as the final stego-image, embedding 1 into pixel (512, 512) by using LSB. Although the PSNR may be lower, the hiding capacity can be greatly improved.

**Figure 7:**
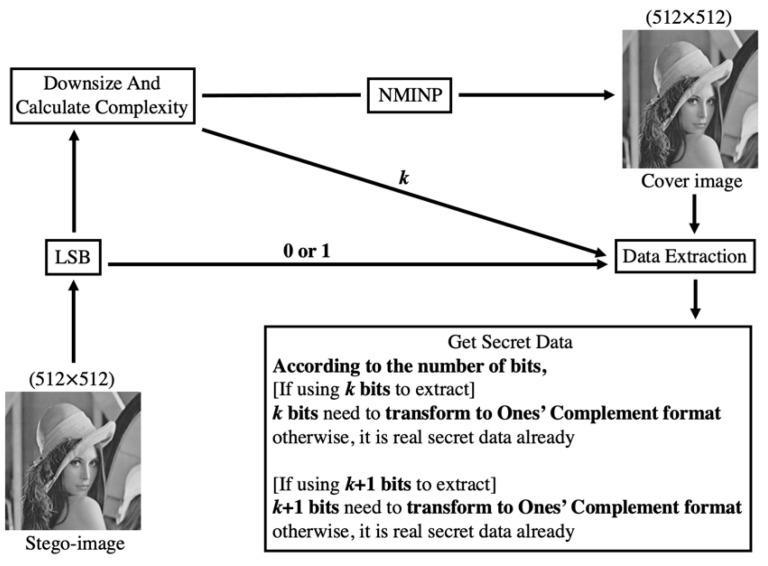
Framework of data extraction in the proposed scheme.

Figure 7 shows the framework of data extraction. First, pixel (512, 512) was extracted using LSB to obtain 0 or 1. If 0 was obtained, each pixel was embedded with at most *k* bits; otherwise, each pixel was embedded with at most *k* + 1 bits. Second, the stego-image was downsized, and the complexity of the downsized image was calculated, where the complexity is denoted as *k*. The cover image was retrieved by using NMINP, from the downsized image. To retrieve the secret data, the absolute value was calculated by subtracting the cover image pixels from the stego-image pixels. After extracting the secret data, if the number of bits is less than *k* or *k* + 1, the data should not be transformed into their ones’ complement format. If the number of bits is equal to *k* or *k* + 1, the data should be transformed into their ones’ complement format to obtain the real secret data. The data extraction process in the proposed method is simple.

### 3.1. Downsize and Calculate Complexity

The original image was downsized to 256 × 256, and the complexity of the downsized image was calculated. The complexity is used to decide the maximum number of bits to be embedded in each pixel, and it is denoted by *k*.

**Figure 8:**
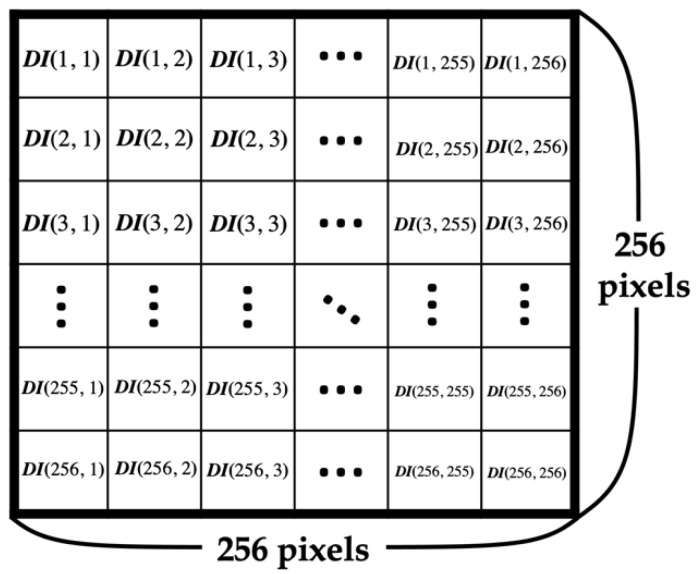
Schematic of the downsized image.

**Figure 9:**
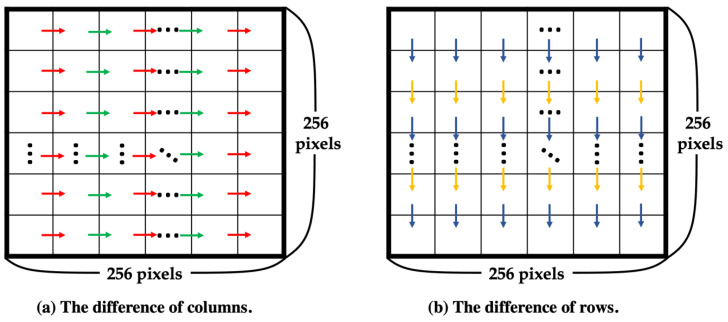
Difference between columns and rows.

**Figure 10:**
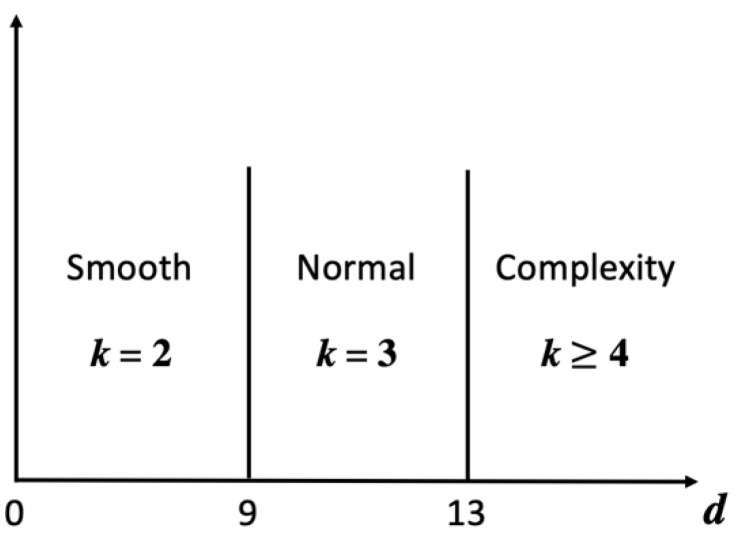
Scope of the complexity (*d*).

Input: Original image (size 512 × 512)
Output: Downsized image (size 256 × 256), *k*
Step 1. Downsize the original image to 256 × 256.
Step 2. To choose the best *k* for a higher PSNR, calculate the complexity of the downsized image using Equations (4)–(6). The pixel of the downsized image is denoted by *DI*(*x*, *y*), as shown in Figure 8, where *x* and *y* range from 1 to 256, and the complexity is denoted by *d*. Equation (4) is used to calculate the difference between columns, denoted by *dColumn*, and Equation (5) is used to calculate the difference between rows, denoted by *dRow*, as shown in Figure 9. Then, sum *dColumn* and *dRow* and divide by 2 to obtain the complexity using Equation (6). After obtaining the complexity value corresponding to the standard in Figure 10, select the best *k*.

(4) $$\mathrm{dColumn}=\frac{\sum_{x=1}^{256}\sum_{y=1}^{255}|\mathrm{DI}\left(x,\;y\right)-\mathrm{DI}(x,\;y+1)|}{256\;\times \;255}$$

(5) $$\mathrm{dRow}=\frac{\sum_{x=1}^{255}\sum_{y=1}^{256}|\mathrm{DI}\left(x,\;y\right)-\mathrm{DI}(x+1,\;y)|}{255\;\times \;256}$$

(6) $$d=\frac{\mathrm{dColumn}+\mathrm{dRow}}{2}$$

### 3.2. NMINP

To improve the HC and PSNR, NMINP does not consider only interpolation (NMI) but also uses the NMI results and takes the original image pixels as a reference to calculate suitable cover image pixels. More details of the NMINP method are as follows.

Input: Original image (size 512 × 512)
Output: Cover image (size 512 × 512)

**Figure 11:**
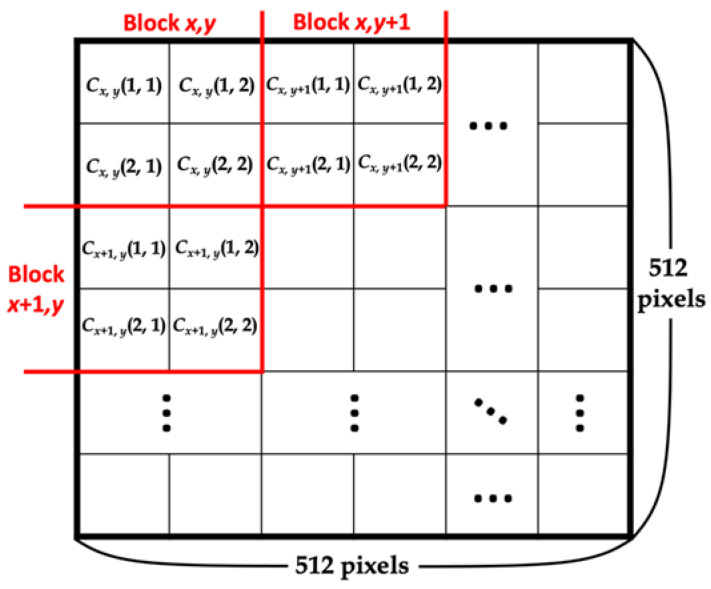
Schematic of dividing the cover image into 2 × 2 non-overlapping blocks and the location of a pixel in the block.

Step 1. Apply interpolation (NMI) to the original image to produce the cover image using Equation (7) and divide the obtained image into 2 × 2 non-overlapping blocks, with each block positioned from left to right and up to down at (1, 1), (1, 2), (2, 1), and (2, 2). For all subsequent equations, the original image pixel is denoted by $O_{x,\;y}(i,\;j)$, the cover image pixel calculated by NMI is denoted by ${\mathrm{NC}}_{x,\;y}(i,\;j)$, where *x* and *y* range from 1 to 255, and (*i, j*) represents the location of the pixel in the block. When calculating the pixels in one block (*x*, *y*), three pixels are used: $O_{x,\;y}(1,1),$ $O_{x,\;y+1}(1,\;1),$ and $O_{x+1,\;y}\left(1,\;1\right)$. Figure 11 shows a schematic of dividing the cover image into 2 × 2 non-overlapping blocks and the location of a pixel in a block.

(7) $$\left\{\begin{array}{c} {\mathrm{NC}}_{x,\;y}(1,1)=O_{x,\;y}(1,1)\\ {\mathrm{NC}}_{x,\;y}(1,2)=[O_{x,\;y}(1,1)+O_{x,\;y+1}(1,1)]/2\\ {\mathrm{NC}}_{x,\;y}(2,1)=[O_{x,\;y}(1,1)+O_{x+1,\;y}(1,1)]/2\\ {\mathrm{NC}}_{x,\;y}(2,2)=[O_{x,\;y}(1,1)+O_{x,\;y+1}(1,1)+O_{x+1,\;y}(1,1)]/3 \end{array}\right.$$

**Figure 12:**
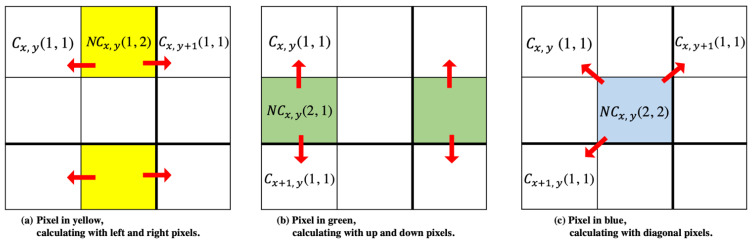
NMI results of pixels in different locations calculated with different neighboring pixels.

Step 2. Based on the NMI results from Step 1, calculate the other suitable cover image pixels, denoted by ${C1}_{x,\;y}(i,\;j)$*,* ${C2}_{x,\;y}(i,\;j)$*,* and ${C3}_{x,\;y}(i,\;j)$*,* using Equation (8). The pixels in different locations calculated with different neighboring pixels are shown in Figure 12.

(8) $$\left\{\begin{array}{c} {C1}_{x,\;y}(i,j)=[{NC}_{x,\;y}(i,j)+C_{x,\;y}(1,1)]/2\\ {C2}_{x,\;y}(i,j)=[{NC}_{x,\;y}(i,j)+C_{x,\;y+1}(1,1)]/2,\mathrm{if}(i,j)=(1,2),(2,2)\\ {C2}_{x,\;y}(2,1)=[{NC}_{x,\;y}(2,1)+C_{x+1,\;y}(1,1)]/2\\ {C3}_{x,\;y}(2,2)=[{NC}_{x,\;y}(2,2)+C_{x+1,\;y}(1,1)]/2 \end{array}\right.$$

Step 3. Based on the results from Steps 1 and 2, calculate the cover image pixel from the original image pixel to determine which result is closer to the original image pixel using Equation (9):

(9) $$\left\{\begin{array}{c} C_{x,\;y}(1,1)=O_{x,\;y}(1,1)\\ C_{x,\;y}(1,2)=\mathrm{argmin}\{|{\mathrm{NC}}_{x,\;y}(1,2)-O_{x,\;y}(1,2)|,|{C1}_{x,\;y}(1,2)-O_{x,\;y}(1,2)|,|{C2}_{x,\;y}(1,2)-O_{x,\;y}(1,2)|\}\\ C_{x,\;y}(2,1)=\mathrm{argmin}\{|{\mathrm{NC}}_{x,\;y}(2,1)-O_{x,\;y}(2,1)|,|{C1}_{x,\;y}(2,1)-O_{x,\;y}(2,1)|,|{C2}_{x,\;y}(2,1)-O_{x,\;y}(2,1)|\}\\ C_{x,\;y}(2,2)=\mathrm{argmin}\{|{\mathrm{NC}}_{x,\;y}(2,2)-O_{x,\;y}(2,2)|,|{C1}_{x,\;y}(2,2)-O_{x,\;y}(2,2)|,|{C2}_{x,\;y}(2,2)-O_{x,\;y}(2,2)|,|{C2}_{x,\;y}(2,2)-O_{x,\;y}(2,2)|\} \end{array}\right.$$

### 3.3. Data-Embedding

The proposed method limits the number of bits to be embedded and occasionally applies ones’ complement. At least one bit of secret data will be embedded in each cover image pixel. After embedding, there are two stego-images: one limits the number of bits embedded in each pixel to at most *k* bits, and the other to at most *k* + 1 bits. By limiting the number of bits to be embedded, the proposed method eliminates the possibility of overflow or underflow. More details of the embedding steps are as follows.

Input: Cover image (size 512 × 512), secret data, *k*, *k* + 1
Output: Two stego-images (size 512 × 512)
Step 1. Calculate the number of bits ($B_{x,\;y}(i,\;j)$) to be embedded in each pixel with the cover and original image pixels using Equation (10). Here, ⌊ ⌋ expresses the floor function. When $C_{x,\;y}(i,\;j)\;–\;O_{x,\;y}(i,\;j)$ is greater than 1, $B_{x,\;y}(i,\;j)$ is equal to log_2_($C_{x,\;y}(i,\;j)\;–\;O_{x,\;y}(i,\;j)$) in the floor function. If $C_{x,\;y}(i,\;j)\;–\;O_{x,\;y}(i,\;j)$ is less than or equal to 1, $B_{x,\;y}(i,\;j)$ is equal to 1.

(10) $$B_{x,\;y}(i,\;j)=\left\{\begin{array}{c} \lfloor log_{2}(|C_{x,\;y}(i,j)–O_{x,\;y}(i,j)|)\rfloor ,\;\mathrm{if}|C_{x,\;y}(i,j)–O_{x,\;y}(i,j)|>1\\ 1,\;\;\;\mathrm{otherwise} \end{array}\right.$$

Step 2 (a). This step is performed for each pixel embedded with a maximum of *k* bits. Check whether the first bit of the embedded secret data is 0 or 1. If the first bit is 0, the result from Step 1, ($B_{x,\;y}(i,\;j)$), is fixed to *k* regardless of its value, and the secret data are embedded in the pixel after performing ones’ complement. If the first bit is 1, the number of bits to be embedded is fixed, and ranges from 1 to *k* – 1. If $B_{x,\;y}(i,\;j)$ is greater than or equal to *k* − 1, the new *B,* denoted by ${B^{\prime }}_{x,\;y}(i,\;j)$*,* is *k* − 1, and the secret data are directly embedded in the pixel. Every secret data bit is transformed into a decimal format before embedding.

$${B^{\prime }}_{x,\;y}(i,\;j)=\left\{\begin{array}{c} k,\;\mathrm{Secret}\;\mathrm{bit}\;\mathrm{begin}\;\mathrm{with}\;0;\\ k-1,\;\mathrm{Secret}\;\mathrm{bit}\;\mathrm{begin}\;\mathrm{with}\;1,\;\mathrm{and}\;B_{x,\;y}(i,j)\geq k-1;\\ B_{x,\;y}(i,j),\;\mathrm{Secret}\;\mathrm{bit}\;\mathrm{begin}\;\mathrm{with}\;1,\;\mathrm{and}\;B_{x,\;y}(i,j)<k-1; \end{array}\right.$$

When ${B^{\prime }}_{x,\;y}(i,\;j)$ = *k*, the secret data should be converted to their ones’ complement format before embedding.

When ${B^{\prime }}_{x,\;y}(i,\;j)$ = 1, the secret data are embedded by LSB.
Otherwise, the cover image pixel is directly added or subtracted from the secret data.

Step 2 (b). This step is performed for each pixel embedded with a maximum of *k* + 1 bits. Check whether the first bit of the embedded secret data is 0 or 1. If the first bit is 0, the result from Step 1, ($B_{x,\;y}(i,\;j)$), is fixed to *k* + 1 regardless of its value, and the secret data are embedded after performing ones’ complement. If the first bit is 1, the number of bits to be embedded is fixed, and ranges from 1 to *k*. If $B_{x,\;y}(i,\;j)$ is greater than or equal to *k*, the new *B,* denoted by ${B^{\prime }}_{x,\;y}(i,\;j),$ is *k*, and the secret data are directly embedded. Every secret data bit is transformed into a decimal format before embedding.

$${B^{\prime }}_{x,\;y}(i,\;j)=\left\{\begin{array}{c} k+1,\;\mathrm{Secret}\;\mathrm{bit}\;\mathrm{begin}\;\mathrm{with}\;0;\\ k,\;\mathrm{Secret}\;\mathrm{bit}\;\mathrm{begin}\;\mathrm{with}\;1,\;\mathrm{and}\;B_{x,\;y}(i,j)\geq k;\\ B_{x,\;y}(i,j),\;\mathrm{Secret}\;\mathrm{bit}\;\mathrm{begin}\;\mathrm{with}\;1,\;\mathrm{and}\;B_{x,\;y}(i,j)<k; \end{array}\right.$$

When ${B^{\prime }}_{x,\;y}(i,\;j)$ = *k* + 1, the secret data should be converted to their ones’ complement format before embedding.

When ${B^{\prime }}_{x,\;y}(i,\;j)$ = 1, the secret data are embedded by LSB.
Otherwise, the cover image pixel is directly added or subtracted from the secret data.

Step 3. Taking the original image pixel as a reference for the cover image pixel to calculate the stego-image pixel using Equation (11), the stego-image pixel is denoted by $S_{x,\;y}(i,\;j)$. If the original image pixel is larger than or equal to the cover image pixel, the cover image pixel is added to the secret data ($D_{x,\;y}(i,\;j)$) to obtain the stego-image pixel; otherwise, subtract the secret data from the cover image pixel to obtain the stego-image pixel.

(11) $$S_{x,\;y}(i,\;j)=\left\{\begin{array}{c} C_{x,\;y}(i,j)+D_{x,\;y}(i,j),\;\mathrm{if}\;O_{x,\;y}(i,j)\geq C_{x,\;y}(i,j);\\ C_{x,\;y}(i,j)–D_{x,\;y}(i,j),\;\mathrm{otherwise}; \end{array}\right.$$

Step 4. Determine whether the stego-image pixel ($S_{x,\;y}(i,\;j)$) overflows or underflows using Equation (12). If the stego-image pixel overflows or underflows, the stego-image pixel is replaced with the cover image pixel.

(12) $$S_{x,\;y}(i,\;j)=\left\{\begin{array}{c} S_{x,\;y}(i,j),\;\mathrm{if}\;0\leq {\;S}_{x,\;y}(i,j)\leq 255;\\ C_{x,\;y}(i,j),\;\mathrm{otherwise}; \end{array}\right.$$

**Figure 13:**
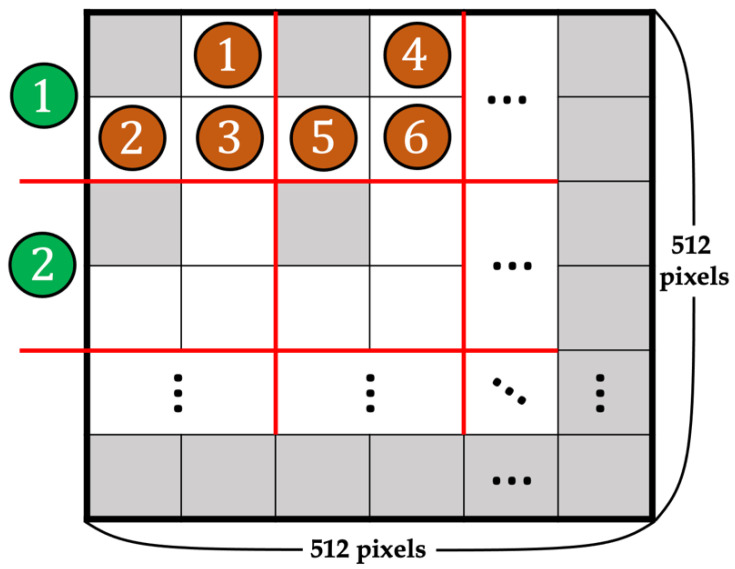
Schematic of embedding order.

Step 5. The order of the block and pixel when embedding is left to right and up to down, respectively. Repeat Steps 1– 4 until all secret data are embedded in all pixels from the 1st column and row to the 511th. The 511th column and row are fixed pixel positions and do not embed any secret data. Figure 13 shows a schematic of the embedding order, where the orange number indicates the order of embedding pixels, and the green number indicates the order of blocks.

### 3.4. Compare the PSNR of Two Stego-Images and Use LSB to Denote Stego-Images Using k or k + 1 for the Most Embedding Bits

To obtain a higher HC, if the difference in PSNR between the two stego-images is not greater than 1.5, the stego-image with *k* + 1 bits as the maximum number of embedding bits will be the final stego-image. This approach increases both the HC and PSNR. In LSB replacement, to record the stego-image, *k* or *k* + 1 bits are used as the maximum number of embedding bits.

Input: Two stego-images (size 512 × 512)
Output: Stego-image (size 512 × 512)
Step 1. The PSNR of the stego-image with *k* bits as the maximum number of embedding bits is denoted by PSNR(S_k). The PSNR of the other stego-image, with *k* + 1 bits as the maximum number of embedding bits, is denoted by PSNR(S_k+1). If the difference between PSNR(S_k) and PSNR(S_k+1) is less than or equal to 1.5, the final stego-image will be the one with *k* + 1 bits as the maximum number of embedding bits.

$$\left\{\begin{array}{c} \mathrm{Stego}-\mathrm{image}(k+1\mathrm{bits}),\;\mathrm{PSNR}(S\_k)-\mathrm{PSNR}(S\_k+1)\leq 1.5;\\ \mathrm{Stego}-\mathrm{image}(k\;\mathrm{bits}),\;\;\;\;\mathrm{otherwise}; \end{array}\right.$$

Step 2. If the final stego-image has *k* + 1 bits as the maximum number of embedding bits, its pixel, S (512, 512), is embedded with “1” by LSB. Conversely, if it has *k* bits as the maximum number of embedding bits, its pixel, S (512, 512), is embedded with “0” by LSB.

### 3.5. Determine the Stego-Images Using k or k + 1 for the Most Embedding Bits

The pixel, S (512, 512), of the stego-image is extracted by using LSB.

Input: Stego-image (size 512 × 512)
Output: 0 or 1
Step 1. The pixel, S (512, 512), of the stego-image is extracted by using LSB. If it is 0, the stego-image is using *k* for the most embedding bits. If it is 1, the stego-image is using *k* + 1 for the most embedding bits.

### 3.6. Downsize Stego-Image and Calculate Complexity

The stego-image is downsized to 256 ×256, and the complexity of the downsized image is calculated. The complexity is used for deciding the maximum number of bits that are going to be embedded in each pixel, and it is denoted as *k*.

Input: Stego-image (size 512 × 512)
Output: Downsized image (size 256 × 256), *k*
Step 1. Downsize the stego-image to 256 × 256.
Step 2. In order to choose the best *k* for a higher PSNR, calculate the complexity of the downsized image by Equations (4)–(6). After obtaining the value of complexity, refer to the standard in Figure 9 to choose the best *k*.

### 3.7. Producing Cover Image before Data Extraction

Input: Downsized image (size 256 × 256)
Output: Cover image (size 512 × 512)
Step 1. Use the proposed method, NMINP, on the downsized image to produce the cover image.

### 3.8. Data Extraction

After producing the cover image, the secret data are extracted from the stego and cover images as follows.

Input: Cover image (size 512 × 512), *k*, 0 or 1
Output: Secret data
Step 1. From the result after using LSB on pixel S (512, 512), 0 means the stego-image used *k* for the most embedding bits, and 1 means the stego-image used *k* + 1 for the most embedding bits.
Step 2. The cover image pixel is subtracted with the stego-image pixel in the absolute value to obtain the secret data in decimal format by using Equation (13). The secret data in decimal denotes as $D_{x,\;y}(i,\;j)$.
  (13) $$D_{x,\;y}(i,\;j)=|C_{x,\;y}(i,\;j)\;–\;S_{x,\;y}(i,\;j)|$$
Step 3. If the stego-image used *k* for the most embedding bits, go to Step 4. If the stego-image used *k* + 1 for the most embedding bits, go to Step 5.
Step 4. From decimal format, convert it to binary format. The number of bits of binary format is denoted as $P_{x,\;y}(i,j)$. If $P_{x,\;y}(i,\;j)$ is equal to *k*, it is converted to its ones’ complement format in order to obtain the real secret data. Otherwise, if $P_{x,\;y}(i,j)$ is equal to 1 to *k* − 1 bits, then it is already the real secret data. If $P_{x,\;y}(i,j)$ is 0, then it means the pixel is overflow or underflow, and it did not embed any secret data.

$$P_{x,\;y}(i,j)=\left\{\begin{array}{c} k,\;\mathrm{convert}\;\mathrm{to}\;\mathrm{its}\;\mathrm{ones}’\;\mathrm{complement}\;\mathrm{format};\\ 0,\;\mathrm{no}\;\mathrm{secret}\;\mathrm{data}\;\mathrm{is}\;\mathrm{embedded}\;\mathrm{in}\;\mathrm{this}\;\mathrm{pixel};\\ \mathrm{otherwise},\;\mathrm{already}\;\mathrm{real}\;\mathrm{secret}\;\mathrm{data}; \end{array}\right.$$

Step 5. From decimal format, convert it to binary format. The number of bits of binary format is denoted as $P_{x,\;y}(i,j)$. If $P_{x,\;y}(i,\;j)$ is equal to *k* + 1, it is converted to its ones’ complement format in order to obtain the real secret data. Otherwise, if $P_{x,\;y}(i,j)$ is equal to 1 to *k* bits, then it is already the real secret data. If $P_{x,\;y}(i,j)$ is 0, then it means the pixel is overflow or underflow, and it did not embed any secret data.

$$P_{x,\;y}(i,j)=\left\{\begin{array}{c} k+1,\;\mathrm{convert}\;\mathrm{to}\;\mathrm{its}\;\mathrm{ones}’\;\mathrm{complement}\;\mathrm{format};\\ 0,\;\mathrm{no}\;\mathrm{secret}\;\mathrm{data}\;\mathrm{is}\;\mathrm{embedded}\;\mathrm{in}\;\mathrm{this}\;\mathrm{pixel};\\ \mathrm{otherwise},\;\mathrm{already}\;\mathrm{real}\;\mathrm{secret}\;\mathrm{data}; \end{array}\right.$$

Step 6. Repeat Step 2 until all the secret data have been extracted. The order of the block and pixel when extracting the secret data is same as for embedding.

### 3.9. Example of Data-Embedding and Extraction

**Figure 14:**
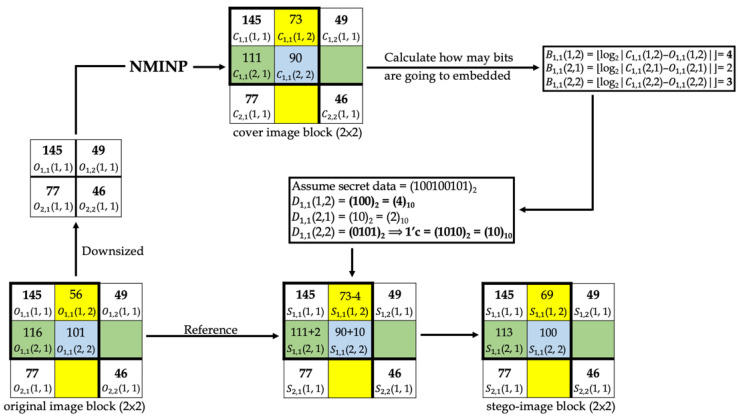
An example of data-embedding.

**Figure 15:**
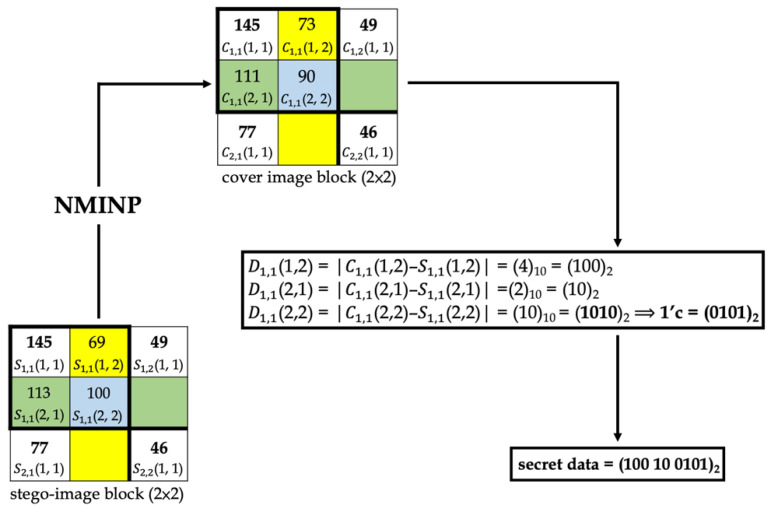
An example of data extraction.

Figure 14 and Figure 15 show examples of data-embedding and extraction, with *k* equal to 4, respectively. The examples use 2 × 2 non-overlapping blocks. The colored pixels embed the secret data, and the pixels with the same color applied the same embedding method.

In Figure 14, first, the original image block was downsized, and the proposed method “NMINP” was applied to produce the cover image pixels using Equations (7)–(9). Then, the number of bits to embed was calculated using the absolute value of the difference between the cover and original image pixels using Equation (10). Afterward, whether the first bit of the secret data to be embedded is 0 or 1 was determined. In $D_{1,\;1}(1,\;2)$, the first bit is 1, and $B_{1,\;1}(1,\;2)$ equals 4; however, when the first bit of the secret data is 1, each pixel embeds at most 3 bits. Therefore, $D_{1,\;1}(1,\;2)$ can embed only 3 bits at most. In $D_{1,\;1}(2,\;2)$, the first bit is 0, and it should embed 4 bits regardless of the result of $B_{1,\;1}(2,\;2)$. The secret data were converted to their ones’ complement before embedding. Finally, the original image pixels were taken as a reference, and if the original image pixels were larger than or equal to the cover image pixels, both pixels were added to obtain the stego-image pixels. Conversely, if the original image pixels were smaller than the cover image pixels, they were subtracted from each other to obtain the stego-image pixels using Equation (11). Equation (12) was used to check whether the stego-image pixel was overflow or underflow, and if it is overflow or underflow, the stego-image pixel will be replaced by the cover image pixel. The last step was to note whether the stego-image was using *k* or *k* + 1 bits for the most embedding bits. We assumed this example uses *k* bits, then embedded 0 into S (512, 512) by LSB.

In Figure 15, first, the cover image pixels were calculated using NMINP. Then, the absolute value of the difference between the cover and stego-image pixels was calculated using Equation (13). The difference will be the secret data in a decimal number format. Next, the decimal format was converted to binary format. $D_{1,\;1}(1,\;2)$ and $D_{1,\;1}(2,\;1)$ indicated 3 and 2 bits in binary format, respectively; thus, they are real secret data. $D_{1,\;1}(2,\;2)$ indicated 4 bits in a binary format, and then it was converted to its ones’ complement to obtain the real secret data. If the difference was 0, the pixel did not embed any secret data.

## 4. Experimental Result

**Figure 16:**
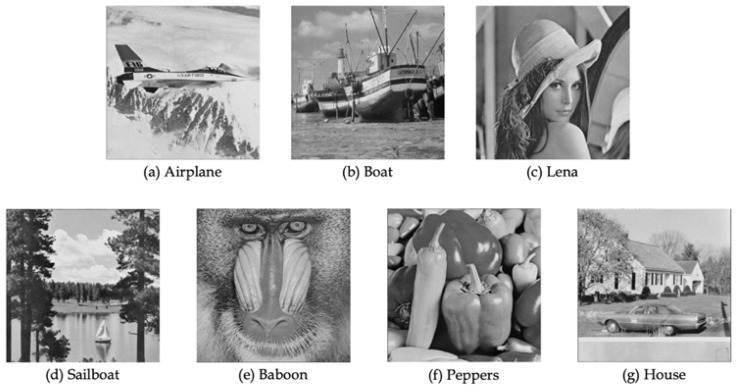
Seven original images.

**Figure 17:**
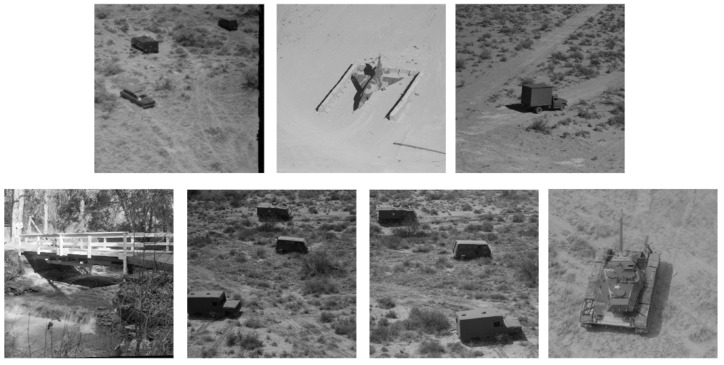
Seven images from the USC-SIPI image database.

In the experiment, several tests were performed using common 512 × 512-sized grayscale images, including “Airplane”, “Boat”, “Lena”, “Sailboat”, “Baboon”, “Peppers”, and “House”, as shown in Figure 16. Additionally, several tests were performed using the other seven 512 × 512-sized grayscale images, as shown in Figure 17. These seven images were all from the USC-SIPI image database. The secret data were generated using a pseudo-random number generator. The proposed method was compared with other methods in terms of HC and PSNR. The result of the structural similarity index (SSIM) was used to compare the original image and the stego-image, as shown in Table 2. SSIM is a standard for comparing whether the original image and the stego-image are similar. From the point of image composition, it defines structural information independent of contrast and brightness, and takes structural information between adjacent pixels into account. The value of SSIM is between 0 and 1. If the value is closer to 1, it means the two images are similar, and if it is closer to 0, the two images are completely dissimilar. In the results, Lena obtained 0.9547, so Lena’s original image and stego-image were very similar. Other images obtained approximately 0.8, so their original image and stego-image were also quite similar. HC refers to all secret bits that can be embedded in the cover image. PSNR is the visual quality of the stego-image compared with the original image. The detailed definitions of HC and PSNR are shown in Equations (14) and (15), respectively:

(14) $$\mathrm{PSNR}=10\log_{10}\;(\frac{{\left(255\right)}^{2}}{\mathrm{MSE}}),$$

(15) $$\mathrm{MSE}=\frac{1}{M\;\times \;N}\sum_{i=1}^{M}\sum_{j=1}^{N}[O(i,\;j)-S(i,\;j)],$$

where 1 ≤ *i* ≤ *M*, 1 ≤ *j* ≤ *N*, and the original image pixel and the stego-image pixel are denoted as O(i, j) and S(i, j), respectively.

(16) $$\mathrm{SSIM}=\frac{\left(2\mu_{x}\mu_{y}+c_{1})(2\sigma_{\mathrm{xy}}\;+c_{2}\right)}{\left(\mu_{x^{2}}+\mu_{y^{2}}+c_{1})(\sigma_{x^{2}}+\sigma_{x^{2}}+c_{2}\right)},$$

where *μ*_x_ and *μ*_y_ are the mean pixel numbers, *μ*_x_^2^ and *μ*_y_^2^ are the variances, and *σ*_x_^2^ and *σ*_y_^2^ are the standard deviations, of the original image and the stego-image, respectively. Here, 2*σμ*_x_*μ*_y_ is the covariance of the original image and the stego-image. Note that *c*_1_ and *c*_2_ are constants, where *c*_1_ = _b1k_ and *c*_2_ = _b2k_. The value of _k_ is 255, b_1_ is 0.01, and b_2_ is 0.03.

Table 3 displays the results of HC and PSNR with different maximum numbers of bits (*k*) embedded. With different *k*, the results highlight in bold are the best result. The results show that if *k* was larger, the HC was higher. However, PSNR was always the best when *k* was smaller. Four images: “Boat”, “Sailboat”, “Peppers”, and “House”, had the best PSNR when *k* was equal to 3, and “Baboon” had the best PSNR when *k* was equal to 4. However, according to the results, “Airplane” and “Lena” had the best PSNR at a small *k*, specifically when *k* was equal to 2.

**Figure 18:**
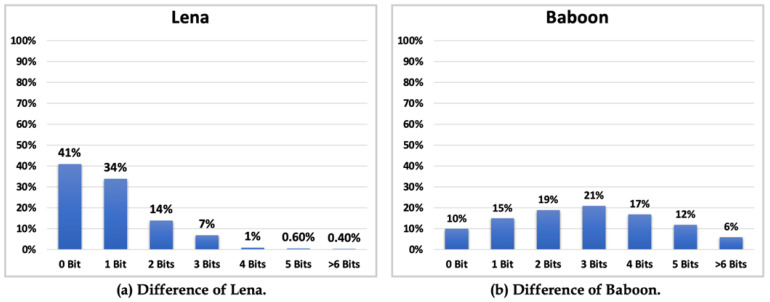
The difference between the cover image pixel and the original image pixel.

Taking “Lena” and “Baboon” as examples, the percentage of the difference between the cover and original image pixels is shown in Figure 18. The difference between these pixels for “Lena” is shown in Figure 18a, whereas that for “Baboon” is shown in Figure 18b. Here, 0 bit means the cover image pixel was the same as the original image pixel, and thus, the difference was 0, while 1 bit means the difference was 1, 2 bits means the difference was 2 or 3, 3 bits means the difference was 4–7, 4 bits means the difference was 8–15, 5 bits means the difference was 16–31, and 6 bits means the difference was 17–63. The method for obtaining the best PSNR is to embed the most secret data into the bit with the highest percentage, but this only occurs in the ideal situation.

According to Table 3 and Figure 18, if the maximum number of bits (*k*) is set to *h* + 1 and the bit with the highest percentage is denoted as *h*, the best result can be obtained when the first bit of the secret data is 1 for each embedding, because the data can directly embed *h* bits. It is not necessary to convert the first bit to its ones’ complement format before embedding, with a fixed number of *h* + 1 bits—this is the perfect situation. Another perfect situation is setting the maximum number of bits (*k*) as *h*, where the best result can be obtained when the first bit of the secret data is 0 for each embedding, because the data need to be converted to their ones’ complement format before embedding, with a fixed number of *h* bits. In reality, perfect situations will not occur because secret data are generated using a pseudo-random number generator, and the frequency of the occurrence of bits 1 and 0 cannot be controlled. Some pixels may slightly differ in terms of the number of bits embedded, but the proposed method has a fixed number of embedding bits, requiring each pixel to embed bits beyond the difference or, in some cases, embed a number of bits less than the difference. These factors lower the PSNR. Another method for obtaining the best PSNR is by calculating the capacity of the image. If the capacity is lower, use a smaller *k*; otherwise, use a larger *k*. The average of PSNR and HC for the 7 images from USC-SIPI image database (Figure 17) was 30.57 and 348,676, respectively.

A comparison of the results is shown in Table 4, including the results of Jung and Yoo [13], Malik et al. [18], and Chen et al. [16], compared with the proposed scheme based on the same image. The best result shows in bold. The results show that the hiding capacity and PSNR of the proposed method were both higher than the other three methods. To obtain a better hiding capacity than the other methods, the proposed method forced embedding of at least 1 bit of secret data into each pixel, and we used ones’ complement in the method, meaning there was no need to use the location map to record whether the first bit of secret data was 0 or 1. In the proposed method, two stego-images were generated by different maximum numbers of bits when embedding, and we obtained two different values of PSNR. If the difference of the two values of PSNR was smaller than 1.5, the stego-image that used *k* + 1 for the maximum number of bits when embedding will be the final stego-image. This dynamic method can greatly improve the embedding capacity and achieve a good PSNR, and the image can maintain high quality, which is the main difference between the proposed method and the other methods.

### Security Analysis

In this section, the proposed method against chi-square steganalysis is presented. The chi-square attack was designed to detect the security of the image after embedding secret data compared with the probability analysis of the original image to check the difference between them. If the difference is near 0 or equal to 0, it means there is no information inside the image, while if the difference is closer to or equal to 1, then it means there is some information inside the image. In steganography, no one should know or be able to detect that there is secret data hidden in the image, so the difference for a good algorithm that can withstand chi-square attacks should be much closer to 0.

**Figure 19:**
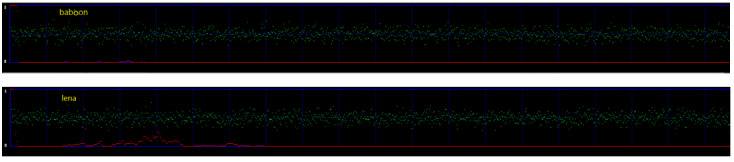
Results of chi-square analysis: Lena and Baboon.

Figure 19 shows the results of the proposed method under the chi-square attack in two stego-images, Lena and Baboon. In order to clearly show the difference, the red line in Figure 19 is flipped horizontally, and it represents the difference. The range of the red line is 0 to 1 to reflect whether the images have information or not. If the red line is closer to 1, it reflects that there is hidden information in the image. This means the method of 1-bit LSB cannot withstand the chi-square attack. If the red line is closer to 0, it reflects that there is no hidden information in the image, and this means the method of 1-bit LSB can withstand the chi-square attack. In the two images, the red line is mostly closer to zero, which means the chi-square attack could not detect any hidden data in these two images. Therefore, the proposed method can withstand the chi-square attacks.

## 5. Conclusions

This paper proposed a NMINP method. The main contribution is that it calculates the cover image pixel based on neighbor mean interpolation (NMI) and neighbor pixels, while using the original image pixel as a reference. Furthermore, our proposed method is dynamic, limiting the number of bits that can be embedded in each pixel to achieve the best PSNR based on the complexity of the downsized image. The experimental result showed that our proposed method outperformed other methods in terms of HC and PSNR, with improvement rates of 20% and 8%, respectively, when embedding at most four bits in each pixel. To obtain a higher HC than the other methods, here, each pixel was forced to embed at least one bit of secret data. The benefits of limiting the number of bits were as follows: it became easier to extract the secret data, the likelihood of overflow or underflow occurring decreased, and a location map was not required to record whether the secret data were flipped to the ones’ complement format.

## Figures and Tables

**Table 1:** Comparison table of other methods and the proposed method.

	Jung and Yoo [13]	Malik et al. [18]	Chen et al. [16]	Proposed Method
Location map	Not needed	Not needed	Needed	Not needed
Dynamic embedding procedure	No	No	No	Yes
When embedding secret data, use original image as a reference	No	No	Yes	Yes
In each 2 × 2 block, how many pixels can be embedded?	3	2	3	3

**Table 2:** The result of SSIM in the proposed method.

Images	SSIM
Airplane	0.8675
Boat	0.8900
Lena	0.9547
Sailboat	0.8854
Baboon	0.8570
Peppers	0.8800
House	0.8700
Average	0.8863

**Table 3:** Hiding capacity (bits) and PSNR (dB) for different *k* values using various images.

**Images**	**HC and PSNR**	***k* = 2**	***k* = 3**	***k* = 4**	***k* = 5**	***k* = 6**
Airplane	HC	267,659	365,121	430,493	501,819	**571,993**
	PSNR	**36.7**	36.48	33.45	27.78	22.48
Boat	HC	267,852	379,064	466,026	539,291	**610,831**
	PSNR	32.93	**33.53**	32.62	28.11	22.05
Lena	HC	267,560	348,070	421,845	491,383	**561,708**
	PSNR	**38.8**	37.76	33.63	27.64	21.63
Sailboat	HC	267,313	383,098	473,004	546,716	**617,698**
	PSNR	33.18	**33.94**	33.10	28.28	22.46
Baboon	HC	267,767	405,046	518,087	609,454	**688,155**
	PSNR	25.93	26.48	**27.19**	26.68	22.29
Peppers	HC	267,504	371,874	452,325	523,592	**592,887**
	PSNR	36.19	**36.66**	34.06	28.25	21.99
House	HC	267,779	366,385	449,509	523,947	**593,681**
	PSNR	33.83	**34.23**	32.80	27.95	22.22
Average	HC	267,633	372,665	458,756	533,743	605,279
	PSNR	33.93	34.15	32.41	27.81	22.16

**Table 4:** Comparisons of hiding capacity (bits) and PSNR (dB).

Comparisons of HC and PSNR					
Images	HC and PSNR	Jung and Yoo [13]	Malik et al. [18]	Chen et al. [16]	Proposed Method
Airplane	HC	358,689	185,676	293,460	**365,121** (*k* = 3)
	PSNR	30.02	30.01	31.43	**36.48**
Boat	HC	355,002	205,368	398,471	**466,026** (*k* = 4)
	PSNR	28.1	28.06	29.47	**32.62**
Lena	HC	325,167	177,777	326,085	**348,070** (*k* = 3)
	PSNR	31.65	31.67	33.27	**37.76**
Sailboat	HC	330,659	205,188	381,576	**473,004** (*k* = 4)
	PSNR	29.07	29.01	30.3	**33.10**
Baboon	HC	309,319	282,272	581,401	**609,454** (*k* = 5)
	PSNR	22.61	22.57	23.7	**26.68**
Peppers	HC	317,769	175,669	327,493	**371,874** (*k* = 3)
	PSNR	29.79	29.78	31.13	**36.66**
House	HC	357,827	214,412	365,111	**449,509** (*k* = 4)
	PSNR	28.45	28.41	29.8	**32.8**
Average	HC	336,347	206,623	381,942	**439,008**
	PSNR	28.52	28.50	29.87	**33.72**

## Data Availability

Not applicable.
